# White Matter Correlates of Theory of Mind in Patients With First-Episode Psychosis

**DOI:** 10.3389/fpsyt.2021.617683

**Published:** 2021-03-05

**Authors:** Nahrie Suk Kim, Tae Young Lee, Wu Jeong Hwang, Yoo Bin Kwak, Seowoo Kim, Sun-Young Moon, Silvia Kyungjin Lho, Sanghoon Oh, Jun Soo Kwon

**Affiliations:** ^1^Department of Brain and Cognitive Sciences, Seoul National University College of Natural Science, Seoul, South Korea; ^2^Biomedical Research Institute, Pusan National University Yangsan Hospital, Yangsan, South Korea; ^3^Department of Psychiatry, Seoul National University College of Medicine, Seoul, South Korea; ^4^Department of Psychiatry, Pusan National University Yangsan Hospital, Yangsan, South Korea

**Keywords:** social cognition, theory of mind, schizophrenia, first episode psychosis, DTI, TBSS, white matter

## Abstract

Deficits in theory of mind (ToM) are considered as a distinctive feature of schizophrenia. Functional magnetic resonance imaging (fMRI) studies have suggested that aberrant activity among the regions comprising the mentalizing network is related to observed ToM deficits. However, the white matter structures underlying the ToM functional network in schizophrenia remain unclear. To investigate the relationship between white matter integrity and ToM impairment, 35 patients with first-episode psychosis (FEP) and 29 matched healthy controls (HCs) underwent diffusion tensor imaging (DTI). Using tract-based spatial statistics (TBSS), fractional anisotropy (FA) values of the two regions of interest (ROI)–the cingulum and superior longitudinal fasciculus (SLF)–were acquired, and correlational analysis with ToM task scores was performed. Among the patients with FEP, ToM strange story scores were positively correlated with the FA values of the left cingulum and left SLF. There was no significant correlation between FA and ToM task scores in HCs. These results suggest that the left cingulum and SLF constitute a possible neural basis for ToM deficits in schizophrenia. Our study is the first to demonstrate the white matter connectivity underlying the mentalizing network, as well as its relation to ToM ability in patients with FEP.

## Introduction

Social cognition abnormalities are core features of schizophrenia (1, 2). Among many aspects of social cognition, deficits in theory of mind (ToM) are consistently reported in patients with schizophrenia across different stages of the disease (3–5). ToM is the mentalizing capacity to infer others' thoughts, intentions, beliefs, and emotions (6). Compared to its severity in other psychiatric disorders, ToM impairment is more severe in schizophrenia (7) and is strongly associated with psychopathology, neurocognitive function, and general functioning (1, 7–11). The impairment has trait-like characteristics that precede the onset of illness and persist across disease progression, even after remission (12–14). Moreover, ToM deficits have been reported as a possible predictor of illness onset among individuals with high-risk psychosis (15). Taken together, these observations suggest the significance of ToM in understanding the pathophysiology of schizophrenia.

Over the last few decades, the neural correlates of ToM have been largely investigated using the functional magnetic resonance (fMRI) approach (16, 17). According to studies in healthy subjects, the mentalizing network, including the medial pre-frontal cortex (mPFC), temporoparietal junction (TPJ), and precuneus/posterior cingulate cortex (PCC), is activated during the performance of various ToM tasks, regardless of task modality (2, 17–20). In schizophrenia, the aberrant activities of the mPFC, TPJ, and precuneus/PCC within the mentalizing network were related to ToM deficits (21–26).

To specify the direct structural connections among the abovementioned ToM regions, white matter studies are necessary. However, there are insufficient number of studies, despite the crucial roles of white matter structures in connecting distal cortical areas (17). Particularly in schizophrenia, only a few studies have attempted to investigate the relationship between white matter and social cognition, such as face perception (27), emotion attribution (28), empathy (29), and social relationships (30). Structural abnormalities that may cause disrupted neural activations and ToM impairment in schizophrenia have not yet been studied.

To explore the white matter neural correlates of ToM deficits in schizophrenia, the cingulum and superior longitudinal fasciculus (SLF) were selected as the white matter regions of interest (ROIs). According to the previous fMRI studies in schizophrenia and DTI literature from healthy individuals and those with other diseases, the two ROIs connect the crucial nodes of mentalizing network as the cingulum passes mPFC and precuneus/PCC and SLF passes through the PFC and TPJ (17, 31–33).

The aim of this study was to investigate the association between ToM deficits and the cingulum and the SLF. With respect to ToM performance, it was hypothesized that patients with FEP would have decreased ToM abilities compared to those of HCs. Additionally, considering previous findings, impaired ToM abilities in FEP patients were hypothesized to be related to the FA reduction in the cingulum and SLF.

## Methods

### Participants

Thirty-five patients with FEP and 29 HCs participated in the study. Age, sex, and handedness were matched between the groups. All participants were part of a prospective cohort study recruited from the psychosis clinic at Seoul National University Hospital. Past and current psychotic symptoms of the patients were evaluated using the Positive and Negative Syndrome Scale (PANSS) (34), and a Structured Clinical Interview for DSM-IV Axis I disorders (SCID-I) was administered. Additionally, Global Assessment of Functioning (GAF) and Hamilton Depression Rating Scale (HAM-D) scores were collected. The inclusion criteria for FEP patients were being aged between 15 and 37 years old with a brief psychotic disorder, schizophreniform disorder, schizophrenia, or schizoaffective disorder. The duration of the illness of all FEP individuals was less than a year. Individuals were excluded from the HC group if they had a past or current SCID-I Non-patient Edition (SCID-NP) axis I diagnosis and any first- to third-degree biological relatives with a psychiatric disorder. The exclusion criteria for both groups were substance abuse, medical illness that could cause psychiatric symptoms, intellectual disability (intelligence quotient [IQ] <70), neurological disorders, or previous head injury. The study procedures were explained in detail to all participants, who then provided written informed consent. This study was approved by the Institutional Review Board of Seoul National University Hospital.

### Behavioral Measures

ToM was assessed with the short form of two verbal ToM tasks: the false belief and strange story tasks (35–38). All tasks were translated into Korean by psychiatrists and clinical psychologists, taking cultural backgrounds into accounts (39). The false belief task consisted of first-order (35) and second-order (36) tasks. The first-order task was used to evaluate whether the subject recognized a character's false belief about reality. The second-order task questioned a character's understanding of the other character's mental state. Each task is comprised of a short vignette with a picture and two questions: one for the comprehension test and the other to assess the subjects' capacity to infer the character's thoughts (justification question). The maximum total score of the false belief task was 12 points.

The strange story task (38) consisted of eight vignettes, each accompanied by a picture and two questions: one for comprehension test and the other to measure subjects' cognitive capacity to infer the character's mental state and emotion in complex naturalistic situations. The task included two examples for each of the four types of stories: double bluff, white lie, persuasion, and misunderstanding. The maximum score of the story task was 26.

### Image Acquisition and DTI Pre-processing

All participants underwent magnetic resonance imaging scanning on a 3T scanner (MAGNETOM Trio Tim Syngo MR B17, 12 channel head coil, Siemens, Erlangen, Germany) at Seoul National University Hospital. Diffusion tensor images were acquired via echo-planar imaging with the following parameters: TR 11400 ms, TE 88 ms, matrix 128 × 128, FOV 240 mm and a voxel size of 1.9 × 1.9 × 3.5. Diffusion-sensitizing gradient echo encoding was applied in 64 directions using a diffusion-weighting factor b of 1,000 s/mm^2^. One volume was acquired with b factor of 0 s/mm^2^ (without gradient).

The diffusion images were pre-processed via three steps with the FSL software package (version 5.0.10; https://fsl.fmrib.ox.ac.uk/fsl/fslwiki/). First, the eddy-current correction was applied to correct distortions and subject movements. Then, the skull was removed by the brain extraction tool (BET). After the BET process, raw brain images underwent visual inspection, and one healthy control was excluded because the dorsal surface of the brain was not covered in the MRI. As the final step, DTIFIT was applied to fit the diffusion tensor model, and individual FA values were obtained.

### Region of Interest

**Figure 1:**
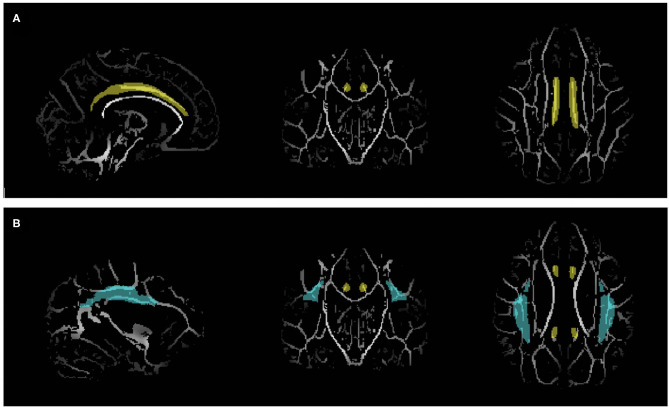
Region of interest (ROI) masks obtained from Johns Hopkins University ICBM-DTI-81 white-matter labels atlas overlaid on the white matter skeleton. **(A)** Cingulum (Yellow). **(B)** Superior longitudinal fasciculus (blue).

To test the structural connectivity among the mentalizing network, two white matter tracts were selected as the regions of interest (ROI). The tracts included the left and right cingulum, which pass through the PFC and precuneus, and the left and right SLF, which pass through the mPFC and TPJ. The ROI masks were obtained from Johns Hopkins University ICBM-DTI-81 white-matter labels atlas (40–42) (Figure 1).

### DTI Processing

Voxel-wise statistical analysis was performed using tract-based spatial statistics (TBSS) in FSL (43, 44). First, a brain mask was generated as a pre-processing step. Then, all subjects' FA images were aligned into a 1 mm × 1 mm × 1 mm Montreal Neurological Institute (MNI) 152 space via FMRIB's Non-linear Image Registration Tool (FNIRT). The aligned images were all merged into a single 4D image file, and the mean FA image was created. A 4D image of the FA skeleton was generated from the mean FA with a threshold of 0.2.

Voxel-wise significant differences between the FEP and HC were investigated using the randomize tool in TBSS. Before processing, age, sex, and handedness were demeaned and fed into the design matrix and contrast file as covariates. The randomize was carried out with 5,000 permutations and threshold-free cluster enhancement (TFCE). The left and right cingulum and SLF masks were used as ROI masks. The threshold for significance was *p* < 0.05.

### Statistical Analysis

The age, sex, and handedness of the final set of subjects were tested to determine whether the variables matched between the groups. The normality of the ToM task scores (e.g., false belief and strange story) was verified, and the scores were compared between the groups via the Mann-Whitney test.

To explore the correlation between ToM task results and the white matter integrity of ROIs, the individual mean FA of each ROI was acquired from 3D individual skeleton images. The individual images were obtained by splitting the 4D skeleton image, which was created from TBSS analysis. Correlation analyses were performed for each mean FA of the cingulum and SLF for both the left and right sides with the false belief and strange story. All statistical analyses were performed using SPSS, version 25 (IBM, Armonk, N.Y.).

## Results

### Demographic Data

The demographic data of the subjects are presented in Table 1. There were no significant differences in the sex ratio, age, handedness, IQ, and education year between the FEP and HC groups.

**Table 1:** Demographic and clinical characteristics of the subjects.

**Variables**	**FEP patients**	**HCs**	**Statistics**	
	**(*n* = 35)**	**(*n* = 28)**	**χ^2^, *F* or *t***	***p***
Age (years)	23.40 ± 5.76	21.68 ± 3.48	−0.612	0.543
Sex (male/female)	16/19	15/13	0.384	0.535
Handedness (right/left)^†^	30/5	26/2	0.804	0.370
IQ	98.11 ± 13.94	100.50 ± 10.63	0.748	0.458
Education (year)	13.26 ± 2.02	13.79 ± 1.89	−1.187	0.235
Parental SES score	2.71 ± 0.86	2.79 ± 0.63	4.28	0.233
PANSS total	68.54 ± 12.00			
PANSS positive	16.23 ± 4.61			
PANSS negative	17.63 ±4.64			
PANSS general	34.69 ± 6.80			
GAF	47.8 ± 11.31			
HAM-D	10.54 ± 4.83			
Olanzapine equivalent dose of antipsychotics (mg/day)	9.07 ± 8.54			

*Data are given as the mean ± S.D*.

† *Classified using Annett Hand Preference Questionnaire*.

*FEP, first-episode psychosis; HC, healthy control; SES, socioeconomic status; PANSS, Positive and Negative Syndrome Scale; GAF, Global Assessment of Functioning; HAM-D, Hamilton Depression Rating Scale*.

### Theory of Mind Task Scores

**Figure 2:**
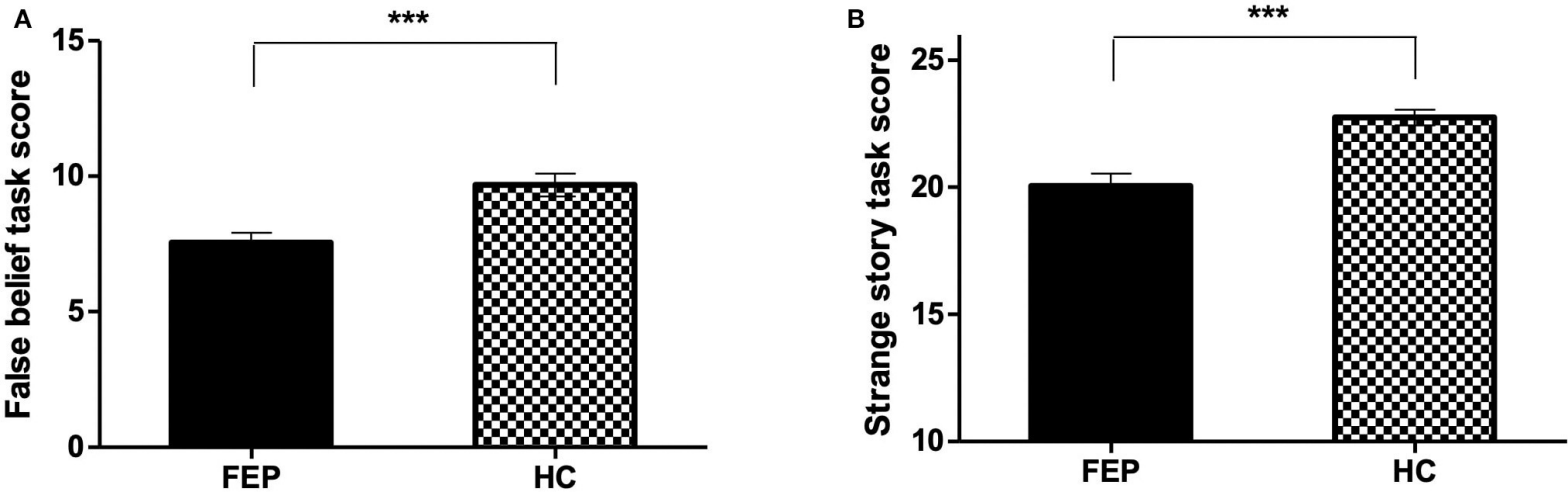
Individual scores of theory of mind tasks (false belief task, strange task) tested using Mann-Whitney. **(A)** False belief scores of FEP patients and HCs (*Z* = −3.506, *p* < 0.001). **(B)** Strange story scores of FEP patients and HCs (*Z* = −4.049, *p* < 0.001). FE, first-episode psychosis; HC, healthy control. ***P ≤ 0.001.

The Mann-Whitney test revealed significant group differences in the two ToM task scores (Table 2). FEP patients exhibited significantly worse performance than HCs in the false belief task (*z* = −3.506, *p* < 0.001) and strange story task (*z* = −4.049, *p* < 0.001). The ToM task results are presented in Figure 2.

**Table 2:** Theory of mind task results.

**Variables**	**FEP patients**	**HCs**	**Statistics**	
	**(*n* = 35)**	**(*n* = 28)**	***Z***	***p***
False belief task	7.54 ± 2.24	9.68 ± 2.21	−3.506	0.000
Strange story task	20.06 ± 2.84	22.75 ± 1.65	−4.049	0.000

*Data are given as the mean ± S.D*.

*FEP, first-episode psychosis; HC, healthy control*.

### TBSS Data and Correlations

**Figure 3:**
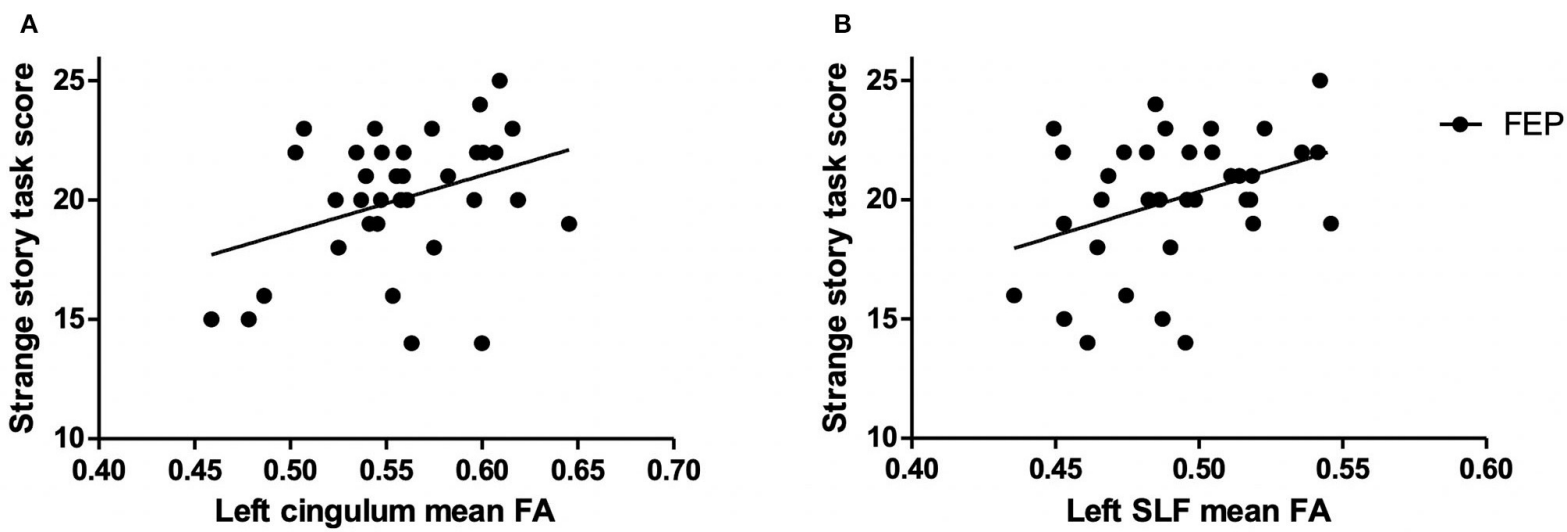
Correlations between the mean fractional anisotropy (FA) values and the strange story task score in first-episode psychosis (FEP) patients. **(A)** The mean FA of the left cingulum and the strange story score (*r* = 0.350; *p* = 0.039). **(B)** The mean FA of the left superior longitudinal fasciculus (SLF) and the strange story score (*r* = 0.374; *p* = 0.027).

TBSS analysis showed no significant voxel-wise difference in any of the ROI regions between FEP and HC groups. To explore the relationship between ToM and white matter integrity, correlational analyses were performed. The results, presented in Figure 3, showed a significant positive correlation between the FA value of the left cingulum and strange story task scores in FEP patients (*r* = 0.35, *p* = 0.039). Also, a significant positive correlation was observed between the FA value of the left SLF and the scores of the strange story task (*r* = 0.374, *p* = 0.027) in FEP patients. No correlation was found between the FA values and false belief scores in FEP patients. There was no significant correlation among the FA values and any ToM task in HCs.

## Discussion

The present study was designed to explore the structural basis of theory of mind (ToM) deficits in schizophrenia. To the best of our knowledge, this is the first attempt to demonstrate the relationship between white matter integrity and ToM in first-episode psychosis (FEP). The results of the false belief task and strange story task showed that the patients with FEP had impaired ToM abilities. The correlation analysis revealed positive associations between the integrity of left ROIs and ToM deficits in FEP patients. However, this correlation was not observed in the healthy controls (HCs). These findings underscore the crucial roles of the left cingulum and left SLF as the structural basis of impaired ToM abilities in FEP.

### ToM Abilities in FEP

The ToM task results were consistent with previous studies that showed decreased ToM performance in FEP patients (5, 45, 46). In this study, two verbal ToM tasks–the false belief task and strange story task–were conducted. The false belief task is the most heavily researched ToM task and is used to assess participants' ability to understand others' mental states, such as thoughts and intentions. The strange story task, invented to measure higher-order ToM, is employed to evaluate the ability to infer not only the thoughts or intentions but also the emotions of others in complex naturalistic situations (38). In the false belief task, FEP patients scored significantly lower than healthy controls, suggesting that the patients have an impaired ability to recognize others' mental states. Similar to the false belief results, the strange story scores of FEP patients were also significantly lower than those of HCs, suggesting the difficulties patients have in inferring others' mental states and emotions in complex situations. These results are in line with previous findings showing ToM deficits as a distinctive feature of schizophrenia, which is found not only in chronic patients but also in the early course of the disease and even in remission (12, 14, 47). As previous meta-analysis studies have suggested, these ToM impairments may be related to the functional outcome of the patients and could be able to predict prognosis (1, 9, 11).

### White Matter Integrity and ToM Abilities

The two ROIs–the cingulum and SLF–were selected based on the atlas-listed white matter tracts that are reported to connect the nodes of the mentalizing network; the mPFC, TPJ, and precuneus/PCC. Our results suggest that the integrity of these white matter ROIs are positively correlated with the strange story task performance in FEP. The correlations were maintained in significant level (*p* < 0.05) even after controlling age and olanzapine equivalent antipsychotic doses (Supplementary Tables 3, 5). This finding is in line with recent DTI studies in healthy children and other patient groups reporting that ToM abilities were associated with the integrity of the cingulum and SLF (31–33, 48).

The cingulum is an association fiber tract that starts from the mPFC and passes through the PCC and precuneus. More precisely, a probabilistic tractography study has revealed that among the listed tracts in the atlas, the cingulum overlaps with 62.59% of the dorsomedial pre-frontal cortex (dmPFC)–PCC fibers and 92.01% of the ventromedial pre-frontal cortex (vmPFC)–PCC fibers (17). The cingulum is known to be involved in attention, memory, and emotional processing (49, 50) and has been postulated as a major white matter tract comprising the mentalizing network (31). In schizophrenia, decreased FA in the cingulum has been reported in both chronic and FEP patients (51–55) and has been linked to both positive and negative symptoms and decreased neurocognition (56, 57).

The SLF is a large association fiber bundle that connects the parietal, occipital and temporal lobes with the frontal cortex (58, 59). The SLF is a core structure involved in attention, memory, language, and emotions (60–63). According to a probabilistic tractography study, 45% of fibers between the dmPFC and TPJ overlap with the SLF (17). Similar to the cingulum, abnormalities in the SLF have been observed in schizophrenia (64–66) and in association with symptoms and neurocognition (67, 68). Along with our results, the abovementioned observations provide converging evidence that the cingulum and SLF are key structures underlying the symptoms and both social and non-social cognitions of schizophrenia.

In this study, only the strange story task scores that reflect higher order ToM were correlated with white matter integrity in FEP patients. According to previous research, complex and higher-order ToM continues to mature until adulthood (47, 69, 70). Several imaging studies have demonstrated neural activations and brain structures related to higher-order ToM change throughout adolescence (71–73). This evidence suggests that the correlation between strange story scores and white matter may reflect an abnormal developmental trajectory of higher-order ToM and related white matter structures in schizophrenia. These findings corroborate the neurodevelopmental hypothesis of schizophrenia (74, 75) and could offer insight into the pathophysiology of the disease, especially focusing on the prodromal stage and early phase.

In contrast to the results of FEP patients, no correlation between the ToM task scores and WM integrities were found in HCs. There can be several reasons for this. First, the homogeneity of the HC group could have led to smaller intragroup variations of the task scores. It is known that age and education level are associated with theory of mind ability (76) but those factors were strictly controlled in this study. Second, it is possible that HCs are less dependent on the direct white matter pathways connecting the mentalizing network regions. Instead, they could use various direct and indirect structural connections among distal brain regions to subserve ToM ability since their neural structures are relatively intact compared to schizophrenia patients. Conversely, schizophrenia patients may utilize large white matter bundles such as cingulum and SLF for ToM process due to the extensive neural degeneration. To specify the association of white matter structures and ToM ability in healthy participants, further research is needed with other ToM task modalities, such as “reading the mind in the eyes” test and subjects with different conditions.

Another finding of the study is that only the left ROIs showed correlation with the ToM task scores in FEP patients. This result matches with the studies of children with traumatic brain injury and patients with surgical resection for diffuse low-grade glioma that reported associations between the left cingulum and ToM abilities (33, 77). These observations suggest that the key white matter structures underlying ToM deficits in FEP may be lateralized to the left hemisphere. However, several studies have indicated that the bilateral or right cingulum is related to ToM abilities (31, 78). Such inconsistency is also evident in findings related to the SLF (32, 48, 78–81).

Despite the inconsistent results, there are studies that have proposed the right SLF may play a crucial role in ToM abilities, while the left contributes mainly to language processing (78, 82). This argument is in contrast to our findings, which showed a relation between the left SLF and ToM ability in FEP patients. There are several possible explanations for this discrepancy. First, different subject groups may result in different outcomes. The “right social brain” argument was developed based on studies of healthy participants and brain damaged patients. However, multiple fMRI studies in schizophrenia and FEP patients have reported bilateral abnormalities in ToM networks (21, 83, 84). This observation suggests that the association between white matter and ToM in schizophrenia may be different than that in other groups.

In addition, the laterality difference between other subject groups and FEP patients may reflect Crows' lateralization hypotheses of schizophrenia (85). Numerous studies have indicated reduced or altered asymmetry in both brain functional connectivity and structures in schizophrenia patients (86, 87), and such alterations are also found in FEP patients (68, 85). These abnormalities in brain asymmetry and functional outcomes are related to developmental problems and the laterality changes in accordance with age (88). Therefore, factors such as the age of onset are closely related to abnormal brain asymmetry (89). Along with the variance in ToM task modalities and heterogeneity among FEP groups, altered or reduced brain asymmetry may have affected the results differently. Since this is the first attempt to investigate the specific white matter structure underlying ToM deficits in schizophrenia, future studies will be necessary to provide reliable evidence on the structural basis of ToM impairment.

### Limitations

Several limitations must be taken into consideration when interpreting the present results. First, no significant differences in FA values between FEP patients and HCs were found in this study. Several DTI studies have reported that the arcuate fasciculus, SLF, and cingulum are intact in FEP patients (90–92), while others have reported white matter alterations in the SLF and cingulum (54, 65). Such inconsistencies may reflect the heterogeneity of the FEP subjects. Symptoms, medications, age of onset, treatment intervention, and other factors may affect white matter changes differently. Additionally, if abnormal structures in FEP patients were located in small sub regions within the cingulum or SLF, the ROI-based TBSS approach would not be able to detect these abnormalities. Since the cingulum and SLF are widely distributed fiber tracts connecting various distal brain regions, averaging the FA values across the whole ROI risks losing some valuable information. To address these issues and to define precise fiber tracts related to impaired ToM in FEP, a probabilistic tractography analysis while controlling the covariates would be necessary in future studies. Second, unlike previous studies the FEP patients in this study were on antipsychotics at the time of scanning and ToM measurements. Antipsychotics are known to be associated with structural alteration in brain (93). However, there was no correlation between olanzapine equivalent antipsychotic doses of the FEP patients and their FA values of white matter ROIs in this study (Supplementary Table 2). Also, the additional partial correlational analysis revealed that the positive correlations between higher order ToM and left cingulum and left SLF were maintained in significant level (*p* < 0.05) even after controlling the effect of antipsychotics. Although, given the previous studies that antipsychotics are associated with white matter alteration (94), it may be beneficial to investigate the relationship between white matter and ToM in drug-naïve FEP patients in future studies.

## Conclusion

Our study was the first to demonstrate the association between FA values in two white matter tracts, the cingulum and the SLF, and ToM ability in FEP patients. This white matter study using DTI methods extends our insight into the neural basis of ToM and suggests that the left cingulum and SLF are vital structures underlying the impairment of ToM in schizophrenia.

## Data Availability Statement

The raw data supporting the conclusions of this article will be made available by the authors, without undue reservation.

## Ethics Statement

The studies involving human participants were reviewed and approved by Academic Affairs Institutional Review Board Seoul National University Hospital. Written informed consent to participate in this study was provided by the participants' legal guardian/next of kin.

## Author Contributions

NK: conceptualization, methodology, software, validation, formal analysis, investigation, data curation, writing–original draft, writing–review and editing, and visualization. TL: conceptualization, methodology, validation, writing–review and editing, and supervision. WH: conceptualization, methodology, and writing–review and editing. YK: software and writing–review and editing. SK: validation, investigation, and writing–review and editing. S-YM: investigation and writing–review and editing. SL: investigation and writing–review and editing. SO: investigation and writing–review and editing. JK: conceptualization, writing–review and editing, resources, supervision, project administration, and funding acquisition. All authors contributed to the article and approved the submitted version.

## Conflict of Interest

The authors declare that the research was conducted in the absence of any commercial or financial relationships that could be construed as a potential conflict of interest.
