# Supplementary material for: White Matter Correlates of Theory of Mind in Patients With First-Episode Psychosis
Source: Front Psychiatry. 2021 Mar 5;12:617683. doi: 10.3389/fpsyt.2021.617683 (PMC7973210; doi:10.3389/fpsyt.2021.617683)
Supplement: Supplementary file 1 [file Data_Sheet_1.PDF]

White matter correlates of theory of mind in patients with first-episode psychosis

Supplemental Materials

Table S1. Correlation results of GAF and HAM-D with RoIs and ToM task scores in FEP

		Left SLF	Left Cingulum	Strange Story score
GAF	Pearson Correlation	.239	-.003	-.033
	Sig.	.168	.988	.849
HAM-D	Pearson Correlation	-.239	-.215	.184
	Sig.	.167	.214	.289

Neither GAF nor HAM-D were correlated with the RoIs or strange story task scores, suggesting that the clinical scores are unlikely the confounding factors in this study.

Table S2. Correlation analysis results between antipsychotics and white matters in FEP

		Left SLF	Left Cingulum
Antipsychotics*	Pearson Correlation	-.079	-.174
	Sig.	.652	.318

*All antipsychotics were converted into olanzapine-equivalent dose (Leucht et al., 2015)

Table S3. Partial correlation analysis results (control variable: antipsychotics) in FEP

		Left SLF	Left Cingulum
Strange story score	Pearson Correlation	.343	.342
	Sig.	.047	.048

Antipsychotics are not correlated with the left cingulum and left SLF (Table S2). Also, the correlation between the white matter RoIs and the high order ToM (strange story tasks) were maintained in significant level even after controlling the effect of antipsychotics (Table S3). These results suggest that the antipsychotics effect is not significant in the results of this study.

Table S4. Correlation analysis results between age and white matter RoIs in FEP

		Left SLF	Left Cingulum
Age	Pearson Correlation	-.063	.237
	Sig.	.721	.171

Table S5. Partial correlation analysis results (control variable: age) in FEP

		Left SLF	Left Cingulum
Strange story task	Pearson Correlation	.378	.348
	Sig.	.028	.043

The left cingulum and SLF are not correlated with age (Table S4). Also, the correlation between the white matter RoIs and the high order ToM (strange story tasks) were maintained in significant level even after controlling age. These results suggest that the effect of age is not significant in this study.
